# Extracorporeal Shock Wave Therapy Reverses Ischemia-Related Left Ventricular Dysfunction and Remodeling: Molecular-Cellular and Functional Assessment

**DOI:** 10.1371/journal.pone.0024342

**Published:** 2011-09-06

**Authors:** Morgan Fu, Cheuk-Kwan Sun, Yu-Chun Lin, Ching-Jen Wang, Chiung-Jen Wu, Sheung-Fat Ko, Sarah Chua, Jiunn-Jye Sheu, Chiang-Hua Chiang, Pei-Lin Shao, Steve Leu, Hon-Kan Yip

**Affiliations:** 1 Division of Cardiology, Department of Internal Medicine, Kaohsiung Chang Gung Memorial Hospital and Chang Gung University College of Medicine, Kaohsiung, Taiwan; 2 Department of Emergency Medicine, E-Da Hospital, I-Shou University, Kaohsiung, Taiwan; 3 Division of General Surgery, Department of Surgery, Kaohsiung Chang Gung Memorial Hospital and Chang Gung University College of Medicine, Kaohsiung, Taiwan; 4 Center for Translational Research in Biomedical Sciences, Kaohsiung Chang Gung Memorial Hospital and Chang Gung University College of Medicine, Kaohsiung, Taiwan; 5 Department of Orthopedic Surgery, Kaohsiung Chang Gung Memorial Hospital and Chang Gung University College of Medicine, Kaohsiung, Taiwan; 6 Department of Radiology, Kaohsiung Chang Gung Memorial Hospital and Chang Gung University College of Medicine, Kaohsiung, Taiwan; 7 Division of Thoracic and Cardiovascular Surgery, Department of Surgery, Kaohsiung Chang Gung Memorial Hospital and Chang Gung University College of Medicine, Kaohsiung, Taiwan; 8 Department of Veterinary Medicine, National Pingtung University of Science and Technology, Pingtung, Taiwan; 9 Graduate Institute of Medicine, College of Medicine, Kaohsiung Medical University, Kaohsiung, Taiwan; University of Modena and Reggio Emilia, Italy

## Abstract

An optimal treatment for patients with diffuse obstructive arterial disease unsuitable for catheter-based or surgical intervention is still pending. This study tested the hypothesis that extracorporeal shock wave (ECSW) therapy may be a therapeutic alternative under such clinical situation. Myocardial ischemia was induced in male mini-pigs through applying an ameroid constrictor over mid-left anterior descending artery (LAD). Twelve mini-pigs were equally randomized into group 1 (Constrictor over LAD only) and group 2 (Constrictor over LAD plus ECSW [800 impulses at 0.09 mJ/mm^2^] once 3 months after the procedure). Results showed that the parameters measured by echocardiography did not differ between two groups on days 0 and 90. However, echocardiography and left ventricular (LV) angiography showed higher LV ejection fraction and lower LV end-systolic dimension and volume in group 2 on day 180 (p<0.035). Besides, mRNA and protein expressions of CXCR4 and SDF-1α were increased in group 2 (p<0.04). Immunofluorescence staining also showed higher number of vWF-, CD31-, SDF-1α-, and CXCR4-positive cells in group 2 (all p<0.04). Moreover, immunohistochemical staining showed notably higher vessel density but lower mean fibrosis area, number of CD40-positive cells and apoptotic nuclei in group 2 (all p<0.045). Mitochondrial protein expression of oxidative stress was lower, whereas cytochrome-C was higher in group 2 (all p<0.03). Furthermore, mRNA expressions of MMP-9, Bax and caspase-3 were lower, whereas Bcl-2, eNOS, VEGF and PGC-1α were higher in group 2 (all p<0.01). In conclusion, ECSW therapy effectively reversed ischemia-elicited LV dysfunction and remodeling through enhancing angiogenesis and attenuating inflammation and oxidative stress.

## Introduction

**C**oronary artery disease (CAD), which develops initially from endothelial dysfunction followed by plaque formation and propagation before the development of obstructive syndrome, remains one of the most notorious killers in industrial counties [1], [2], [3]. Despite the advance in pharmacological therapy [4], [5], mature technique of coronary artery bypass grafting (CABG) [6], [7], newly developed instrument-supported percutaneous coronary intervention (PCI) [8], [9] for restoring myocardial perfusion, and current guideline focused on management strategy [10], restenosis due to preexisting systemic endothelial dysfunction and diffused vascular obstruction remain the Achilles' heel that limits therapeutic success and long-term prognostic outcome [11], [12], [13]. Of importance is that quite a lot of patients suffering from diffuse obstructive CAD, for whom pharmacological treatment is of limited help, are not suitable candidates for interventional therapy through either PCI or CABG. The majority of these patients, therefore, are still in a helpless clinical situation. Hence, finding a safe and effective therapeutic regimen for patients who have diffuse obstructive CAD, especially those unsuitable for coronary intervention, is of utmost importance for cardiologists and cardiovascular surgeons.

Shock wave (SW) therapy can deliver a sequence of transient pressure disturbances characterized by high peak pressure (100 MPa), fast pressure rise (<10 ns), rapid propagation, and short lifecycle (10 µs) produced by an appropriate generator and directed to a specific target area with an energy density in the range of 0.003–0.890 mJ/mm^2^ [14] [15], [16], [17]. A number of studies have revealed that not only does SW provide mechanical means of treatment such as in lithotripsy for kidney and ureteral stones, but its low-energy form (0.03 to 0.11 mJ/mm^2^) also produces a series of subtle biological changes in the musculoskeletal [18], [19] and cardiovascular system [15], [16], [17]. Additionally, *in vitro* studies have demonstrated that extracorporeal (EC) SW therapy can enhance vascular endothelial growth factor (VEGF) mRNA expression in cultured human umbilical vein endothelial cells [15] and in rat bone marrow cells (BMCs) [20] as well as promote BMCs differentiation into cells with endothelial phenotype [20]. Hence, by applying appropriate energy to ischemic organs [15] or tissues [18], [21], ECSW therapy can attenuate inflammatory response and induce angiogenesis/vasculognesis [15], [18], [21]. Thus, ESCW therapy may provide promising therapeutic benefits in relieving the ischemic syndrome for patients with diffuse obstructive CAD who are not suitable candidates for interventional therapies. However, prior to utilization of ECSW for daily clinical practice, a pre-clinical experimental model for verifying its safety and efficacy is mandatory. This study utilized a mini-pig ischemic heart model to test the hypothesis that ECSW therapy may improve ischemia-related left ventricular (LV) dysfunction and attenuate LV remodeling.

## Methods

### Ethics

All experimental animal procedures were approved by the Institute of Animal Care and Use Committee at Chang Gung Memorial Hospital – Kaohsiung Medical Center (Affidavit of Approval of Animal Use Protocol No. 2006121501) and performed in accordance with the Guide for the Care and Use of Laboratory Animals (NIH publication No. 85–23, National Academy Press, Washington, DC, USA, revised 1996).

### Animals, Protocol, Procedures

Male mini-pig (Taitung Animal Propagation Station, Livestock Research Institute, Taiwan), weighting 16–18 kg, was anesthetized by intramuscular injections of ketamine (15 mg/kg) and maintained with an inhalation of 1.5% isoflurane during the procedures. After being shaved on the chest, the mini-pig was placed in supine position on a warming pad at 37°C followed by endotracheal intubation with positive-pressure ventilation (180 ml/min) with room air using a ventilator (Sn: Q422ZO, SIMS PneuPAC, Ltd.). Electrocardiographic (ECG) monitor and defibrillator were then connected to the chest wall.

Under sterile conditions, the heart was exposed through mid-thoracotomy. After gentle removal of the pericardium, an ameroid constrictor (Research Instruments NW, Inc. Lebanon, OR, USA) was applied to the mid-left anterior descending artery (LAD) just distal to the first diagonal branch. The constrictor constitutes an outer layer of stainless steel and an inner layer of fluid-absorbing casein that swells as it slowly absorbs body fluid to produce an inward concentric expansion. It usually required several weeks to create a significant stenosis over the mid-portion of LAD.

**Figure 1:**
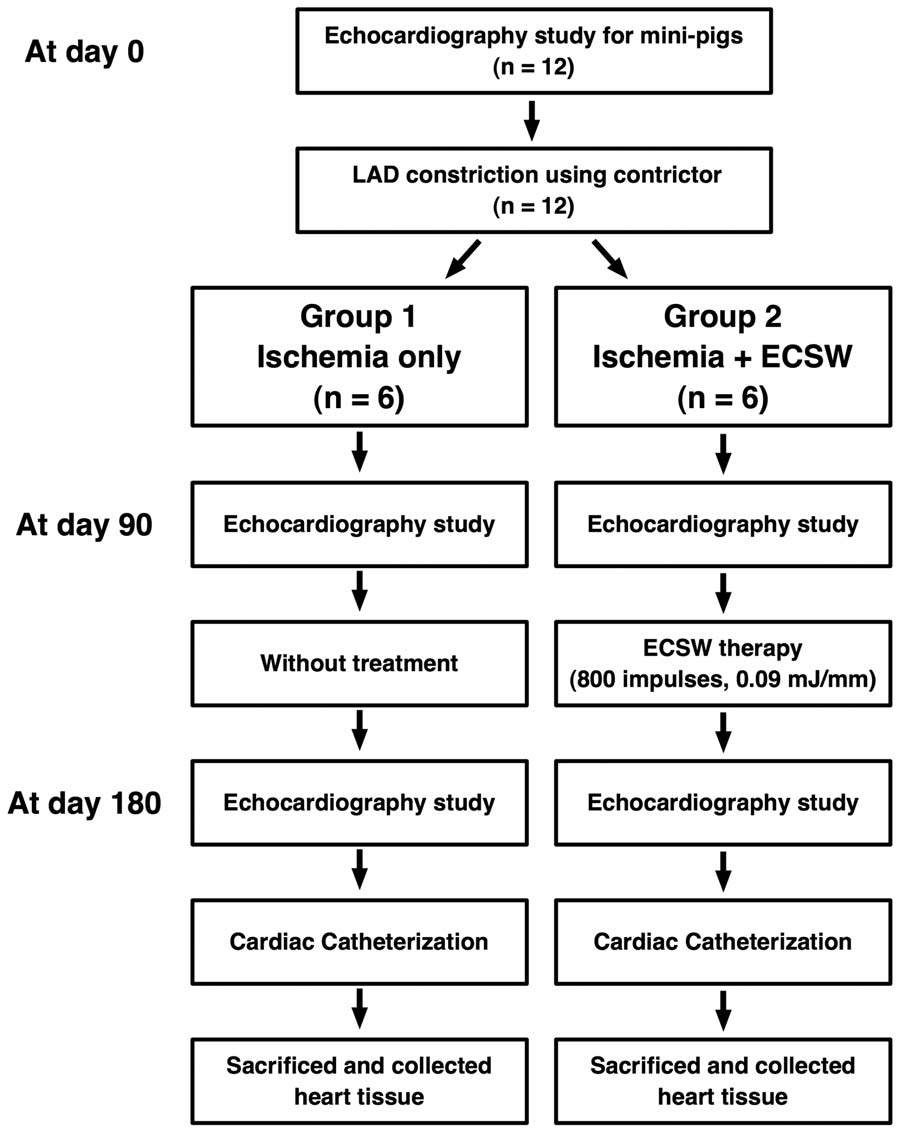
Detailed protocol and procedure. Schematic illustration of the detailed protocol on preparative procedure of extracorporeal shock wave (ECSW) therapy. LAD = left anterior descending artery.

Neither regional ischemia nor wall motion abnormality was noted over LV anterior wall after the procedure. The absence of cardiac ischemia was further confirmed by complete ECG following the procedure. Ameroid constrictors were applied to mid-LAD in twelve mini-pigs which were equally divided into group 1 (Constrictor over LAD only) and group 2 [Constrictor over LAD plus ECSW (800 impulses at 0.09 mJ/mm^2^) after the procedure]. The muscle and skin were then closed in layers. The animals were allowed to remain on the warming pad and recover under care. A flowchart of the experimental procedure is shown in Figure 1.

To compare the findings at cellular-molecular level between normal and disease animals (i.e. groups 1 and 2), standard control samples were obtained from six healthy mini-pigs with similar age and body weight that served as normal controls.

### Application of ECSW to LV Myocardium of Mini-Pig

By day 90 after the procedure, each mini-pig in group 2 was anesthetized again without intubation. ECSW therapy was applied to group 2 animals on day 90 after the procedure using the shock wave generator system (Cardiospec, Medispec, Israel). A cardiac ultrasound imaging system was used to locate the treatment areas of left ventricle. Under transthoracic sonographic guidance, shock waves were then delivered via a special applicator through the anatomical acoustic window to the treatment area under ECG R-wave gating. Briefly, after echocardiographic localization of the four ischemic zones of LV (i.e. middle anterior, middle anteroseptal, middle inferior, and middle inferolateral segments) using parasternal axis approach, ECSW was applied once to each of the four ischemic zones at an intensity of 200 impulses at 0.09 mJ/mm^2^/zone which was a dosage modified according to a previous report [15] so that the total energy applied to LV myocardium was 800 impulses at 0.09 mJ/mm^2^.

### Functional Assessment by Echocardiography

Transthoracic echocardiography was performed preoperatively and on days 90 and 180 after the procedure under general anesthesia as described previously [22] using an ultrasound system (Hewlett Packard SONOS 7500) equipped with 2.5 to 3.5 MHz linear probes. With the animals in a supine position, left ventricular internal dimensions [i.e. end-systolic diameter (ESD) and end-diastolic diameter (EDD)] were measured according to the American Society of Echocardiography leading-edge method using at least 3 consecutive cardiac cycles [22].

The LV ejection fraction (LVEF) was calculated as: LVEF (%)  =  [(LVEDD^3^−LVEDS^3^)/LVEDD^3^] x 100. All measurements were performed by a cardiologist blinded to treatment and non-treatment groups.

### Cardiac Catheterization

By six months following the procedure, cardiac catheterization was performed via right common carotid artery. A 6-French pigtail was used for measuring the arterial blood pressure in ascending aorta, LV systolic and end-diastolic pressure as well as for performing left ventriculography. Coronary angiographic study was achieved using a 6-French Kimny guiding catheter (Boston Scientific, Scimed, Inc. Maple Grove, MN).

Left ventriculography, which was immediately performed after the insertion of arterial sheath into right common carotid artery, was recorded at 30° right anterior oblique and 60° left anterior oblique views. The blood pressures of ascending aorta and LV chambers as well as LVEF were then determined.

The mini-pigs were sacrificed by intra-coronary injection of potassium chloride in catheterization room after the procedure. The heart was carefully removed for further studies.

### Immunohistochemical staining for CD40 and Immunohistofluorescence staining for von Willebrand Factor (vWF)

Paraffin sections (3 µm thick) were obtained from LV myocardium of each mini-pig. To block the action of endogenous peroxidase, the sections were initially incubated with 3% hydrogen peroxide, and then further processed using Beat Blocker Kit (Zymed Company, #50–300) with immersion in solutions A and B for 30 minutes and 10 minutes, respectively, at room temperature. Polyclonal rabbit antibodies against CD40 (dilution 1/100; Spring Bioscience) were then used, followed by application of SuperPicTure^TM^ Polymer Detection Kit (Zymed) for 10 minutes at room temperature. Finally, the sections were counterstained with hematoxylin. For negative control experiments, primary antibodies were omitted.

Immunohistofluorescence (IHF) staining for vWF-positive cells, an index of endothelial cells in capillaries, was performed with anti-vWF antibody (Chemicon).

### Oxidative Stress Reaction of LV Myocardium

The Oxyblot Oxidized Protein Detection Kit was purchased from Chemicon (S7150). The procedure was performed according to our recent study [23]. The 2,4-Dinitrophenylhydrazine (DNPH) derivatization was carried out on 6 μg of protein for 15 minutes according to manufacturer's instructions. One-dimensional electrophoresis was carried out on 12% SDS/polyacrylamide gel after DNPH derivatization. Proteins were transferred to nitrocellulose membranes which were then incubated in the primary antibody solution (anti-DNP 1:150) for 2 hours, followed by incubation with second antibody solution (1:300) for one hour at room temperature. The washing procedure was repeated eight times within 40 minutes. Immunoreactive bands were visualized by enhanced chemiluminescence (ECL) which was then exposed to Biomax L film (Kodak). For quantification, ECL signals were digitized using Labwork software (UVP). On each gel, a standard control sample was loaded.

### Isolation of Mitochondria for Cytochrome C and Connexin (Cx)43

The LV myocardium was excised and then washed with buffer A (100 mM Tris-HCl, 70 mM sucrose, 10 mM EDTA and 210 mM mannitol, pH 7.4). Samples were minced finely in cold buffer A and then incubated for 10 min. All samples were homogenized in an additional 3 mL of buffer A using a motor-driven grinder. The homogenate was centrifuged twice at 700 g for 10 min at 4°C. The supernatant was centrifuged again at 8,500 g for 15 min, and the pellets were washed with buffer B (10 mM Tris-HCl, 70 mM sucrose, 1 mM EDTA, and 230 mM mannitol, pH 7.4). The mitochondria-rich pellets were collected and stored at −70°C.

### Western Blot Analysis for Connexin 43, CXCR4, Stromal Cell-Derived Factor (SDF)-1α, and Cytochrome C in LV Myocardium

Equal amounts (10–30 µg) of protein extracts from remote viable LV myocardium were loaded and separated by SDS-PAGE using 8–10% acrylamide gradients. Following electrophoresis, the separated proteins were transferred electrophoretically to a polyvinylidene difluoride (PVDF) membrane (Amersham Biosciences). Nonspecific proteins were blocked by incubating the membrane in blocking buffer (5% nonfat dry milk in T-TBS containing 0.05% Tween 20) overnight. The membranes were incubated with the indicated primary antibodies (Cx43, 1:1000, Chemicon; CXCR4, 1:1000, Abcam; SDF-1, 1:1000, Cell Signaling; cytochrome C, 1: 1000, BD Biosciences; Actin, 1:10000, Chemicon) for 1 h at room temperature for Cx43, cytochrome C and CXCR4 and overnight at 4°C for SDF-1α, respectively. Horseradish peroxidase-conjugated anti-mouse immunoglobulin IgG (1:2000, Amersham Biosciences) was applied as the second antibody for Cx43 for 1 h at room temperature; Horseradish peroxidase-conjugated anti-rabbit immunoglobulin IgG (1:2000, Cell Signaling) was applied as the secondary antibody for one hour for Cx43, cytochrome C and CXCR4 and 45 minutes for SDF-1α at room temperature. The washing procedure was repeated eight times within 1 h.

### Real-Time quantitative PCR Analysis

Real-time polymerase chain reaction (PCR) was conducted using LighCycler TaqMan Master (Roche, Germany) in a single capillary tube according to the manufacturer's guidelines for individual component concentrations. Forward and reverse primers were each designed based on individual exon of the target gene sequence to avoid amplifying genomic DNA.

During PCR, the probe was hybridized to its complementary single-strand DNA sequence within the PCR target. As amplification occurred, the probe was degraded due to the exonuclease activity of Taq DNA polymerase, thereby separating the quencher from reporter dye during extension. During the entire amplification cycle, light emission increased exponentially. A positive result was determined by identifying the threshold cycle value at which reporter dye emission appeared above background.

### Vessel Density in LV Myocardium

Immunohistochemical (IHC) staining of arterioles was performed with α-SMA (1:400) as primary antibody at room temperature for 1 h, followed by washing with phosphate buffered saline (PBS) thrice. The anti-mouse-horseradish peroxidase-conjugated (HRP) conjugated secondary antibody was then added for 10 min, followed by washing with PBS thrice. The 3, 3’ diaminobenzidine (DAB) (0.7 gm/tablet) (Sigma) was added for 1 minute, followed by washing with PBS thrice. Finally, hematoxylin was added for 1 minute as a counter stain for nuclei, followed by washing twice. Three coronal sections of the heart were analyzed in each mini-pig. For quantification, three randomly selected high-power fields (HPFs) (x200) were analyzed in each slide. The mean number per HPF for each animal was then determined by summation of all numbers divided by 9.

### Histological Study of Fibrosis Area

Masson's trichrome staining was used for studying fibrosis of LV myocardium. Three serial sections of LV myocardium were prepared at 4 µm thickness by Cryostat (Leica CM3050S). The integrated area (µm^2^) of fibrosis in the slides was calculated using Image Tool 3 (IT3) image analysis software (University of Texas, Health Science Center, San Antonio, UTHSCSA; Image Tool for Windows, Version 3.0, USA). Three selected sections were quantified for each animal. Three randomly selected HPFs (400 x) were analyzed in each section. After determining the number of pixels in each fibrotic area per HPF, the numbers of pixels obtained from the three HPFs were summed. The procedure was repeated in two other slides for each animal. The mean pixel number per HPF for each animal was then determined by summating all pixel numbers and dividing by 9. The mean the integrated area (µm^2^) of fibrosis in LV myocardium per HPF was obtained using a conversion factor of 19.24 (1 µm^2^ represented 19.24 pixels).

### TUNEL Assay for Apoptotic Nuclei

For each animal, 6 sections (3 longitudinal and 3 transverse sections of LV myocardium) were analyzed by an *in situ* Cell Death Detection Kit, AP (Roche) according to the manufacture's guidelines. The TUNEL-positive cells was examined in 3 randomly chosen HPF (×400) and normalized to the total number of cells divided by 18.

### Statistical Analysis

Data were expressed as mean values (mean ± SD). The significance of differences between two groups was evaluated with *t*-test. The significance of differences among the groups was evaluated using analysis of variance followed by Bonferroni multiple-comparison post hoc test. Statistical analyses were performed using SAS statistical software for Windows version 8.2 (SAS institute, Cary, NC). A probability value <0.05 was considered statistically significant.

## Results

### Serial Echocardiography Findings and Six-Month Angiographic Findings

There were no significant differences in initial and day 90 LVEDD, LVESD, left ventricular end-diastolic volume (LVEDV), left ventricular end-systolic volume (LVESV), LVEF and FS between group 1 (without ECSW therapy) and group 2 (with ECSW therapy) (Table 1). Moreover, LVEDD and LVEDV were similar between group 1 and group 2 on day 180 after the procedure. However, LVESD (2.97±0.58 vs. 2.24±0.31, p = 0.021) and LVESV (36.0±17.4 vs. 17.2±6.3, p = 0.032) were significantly higher, whereas LVEF (43.7±10.1 vs. 63.9±9.9, p = 0.006) and FS (20.9±5.5 vs. 36.1±7.3, p = 0.002) were significantly lower in group 1 than in group 2 on day 180 after the procedure. Furthermore, using paired two-tailed *t*-test for LVEDD between 90 day and 180 day in the same group, this parameter was found to be significantly reduced in group 2 (4.02±0.46 vs. 3.52±0.48, p = 0.043), but it showed no difference in group 1 (3.70±0.45 vs. 3.73±0.49, p = 0.897). These findings imply that ECSW therapy attenuated ischemia-related LV remodeling and improved ischemia-related LV dysfunction. Of importance is the fact that no ECSW therapy-related side effect was observed.

**Table 1:** Serial Echocardiography Findings and Six-Month Angiographic results of the Animals.

Variables	Group 1*(n = 6)	Group 2*(n = 6)	p-value
Echocardiography (Day-0)			
LVEDD (cm)	3.23±0.43	3.56±1.46	0.090
LVESD (cm)	2.31±0.36	2.59±0.13	0.082
LVEDV (mL)	43.0±13.1	53.5±5.1	0.093
LVESV (mL)	18.9±6.9	24.1±3.4	0.110
FS (%)	28.7±1.9	27.8±0.9	0.268
LVEF (%)	56.6±3.3	54.7±1.5	0.224
Echocardiography (Day-90)			
LVEDD (cm)	3.70±0.45	4.02±0.46	0.866
LVESD (cm)	2.76±0.54	2.71±0.46	0.861
LVEDV (mL)	59.4±15.6	57.5±16.1	0.851
LVESV (mL)	30.1±13.7	28.2±11.0	0.806
FS (%)	25.8±7.1	26.3±4.1	0.798
LVEF (%)	51.5±12.7	52.1±6.9	0.764
Echocardiography (Day-180)			
LVEDD (cm)	3.73±0.49	3.52±0.48	0.474
LVESD (cm)	2.97±0.58	2.24±0.31	0.021
LVEDV (mL)	57.4±21.3	52.2±16.5	0.650
LVESV (mL)	36.0±17.4	17.2±6.3	0.032
FS (%)	20.9±5.5	36.1±7.3	0.002
LVEF (%)	43.7±10.1	63.9±9.9	0.006
Angiographic Results (Day-180)			
LVEF (%)	43.8±9.6	62.5±8.4	0.004
SBP of AsAo (mmHg)	154.2±9.9	141.1±27.0	0.327
DBP of AsAo (mmHg)	123.6±6.20	101.9±22.3	0.06
LVSBP (mmHg)	140.4±5.3	133.5±20.6	0.394
LVEDP (mmHg)	18.6±4.8	8.4±2.7	<0.001
180-day mortality	0% (0)	0% (0)	—
Malignant dysrhythmia	0% (0)	0% (0)	—

Data are expressed as mean ±SD or %.

LVEDD = left ventricular end-diastolic dimension; LVESD = left ventricular end-systolic dimension; LVEDV = left ventricular end-diastolic volume; LVESV = left ventricular end-systolic volume; FS = fraction shortening; LVEF = left ventricular ejection fraction; SBP of AsAo = systolic blood pressure of ascending aorta; DBP = diastolic blood pressure; LVEDP = left ventricular end-diastolic pressure.

*group 1 = ischemia without extracorporeal shock wave (ECSW) treatment; group 2 = ischemia plus ECSW-treated.

Six-month angiographic results showed that the systolic or diastolic blood pressure of ascending aorta and LV systolic pressure were similar between group 1 and group 2. However, LV end-diastolic pressure was remarkably higher (18.6±4.8 vs. 8.4±2.7, p<0.001), whereas LVEF (43.8±9.6 vs. 62.5±8.4, p = 0.004) was substantially lower in group 1 than in group 2 (all p<0.005). These findings further support the effect of ECSW therapy on inhibiting LV remodeling and improving LV function in the setting of myocardial ischemia.

### Protein Expressions of Mitochondrial Oxidative Stress and Cytochrome C in Mitochondria and Cytosol of LV Myocardium

**Figure 2:**
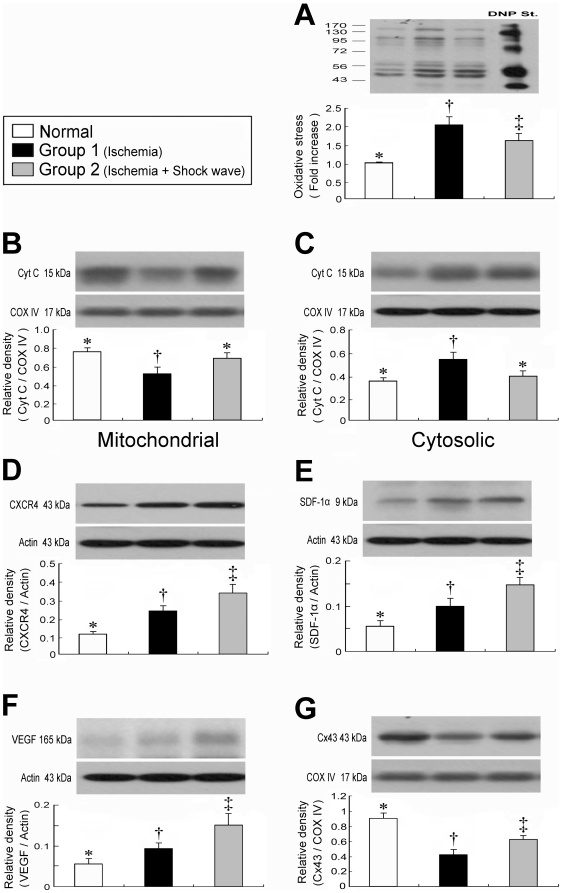
Expression of oxidative stress- and angiogenesis-related proteins in LV myocardium. **A**) Protein expression of oxidative stress. †p<0.001 between the indicated groups. **B**) Protein expression of cytochrome C in mitochondrial level. †p<0.04 between the indicated groups. **C**) Protein expression of cytochrome C in cytosolic level. †p<0.04 between the indicated groups. **D**) Protein expression of CXCR4. †p<0.03 between the indicated groups. **E**) Protein expression of stromal cell-derived factor (SDF)-1α. †p<0.05 between the indicated groups. **F**) Protein expression of VEGF. †p<0.03 between the indicated groups. **G**) Protein expression of connexin43 (Cx43). †p<0.005 between the indicated groups. Symbols (*, †, ‡) indicate significance (at 0.05 level) (by one-way ANOVA and Bonferroni multiple comparison post hoc test, n = 6 in each group).

Compared with normal controls, Western blotting revealed notably enhanced mitochondrial oxidative stress (Fig. 2A) in groups 1 and 2 (all p<0.001). Moreover, the protein expression of oxidative stress in mitochondria was remarkably increased in group 1 than in group 2 (p<0.05). The total cytochrome C protein expression in both mitochondria (Fig. 2B) and cytosol (Fig. 2C) did not differ between normal controls and group 2, whereas its expression in mitochondria was significantly reduced in group 1 as compared with normal controls and group 2 (all p<0.04). In contrast, its cytosolic expression was significantly enhanced in group 1 compared to normal controls and group 2 (all p<0.04). These findings indicate that the expression of cytochrome C, an index of energy supply and storage in mitochondria, was relatively well-preserved in group 2 compared with that in group 1. The increase in cytosolic cytochrome C content also suggests significant mitochondrial damage with cytochrome C release into the cytosol in the myocardium of group 1 animals. These findings highlight the fact that ECSW therapy significantly alleviated the intensity of oxidative stress resulting from free radical production due to a change in permeability of the mitochondrial transition pore after myocardial ischemia.

### Angiogenesis-related protein expression of CXCR4, SDF-1 and VEGF and Cx43 of LV Myocardium

The protein expressions of CXCR4 (Fig. 2D), an index of endothelial progenitor cells (EPCs), and SDF-1α (Fig. 2E), a chemokine for EPCs homing in ischemic tissue, were remarkably higher in group 2 as compared with those in normal controls and in group 1, and notably higher in group 1 than in normal controls (all p<0.05). Moreover, VEGF (Fig. 2F), an index of chemokine for angiogenesis, was notably higher in group 2 than in both normal controls and group 1, and significantly higher in group 1 than in normal controls (all p<0.03). These findings indicate that EPCs, SDF-1α and VEGF were increased in response to ischemic stimuli and ECSW therapy enhanced these cellular and chemokine expressions for angiogenesis which, in turn, leaded to an increase in the number of endothelial cells in ischemic tissue.

The protein expression of Cx43 (Fig. 2G), an indicator of the integrity of signal transduction and communication between cardiomyocytes, was significantly lower in group 1 than in both normal controls and group 2, and was notably lower in group 2 than in normal controls (all p<0.005).

### Apoptosis and Fibrosis of LV Myocardium

**Figure 3:**
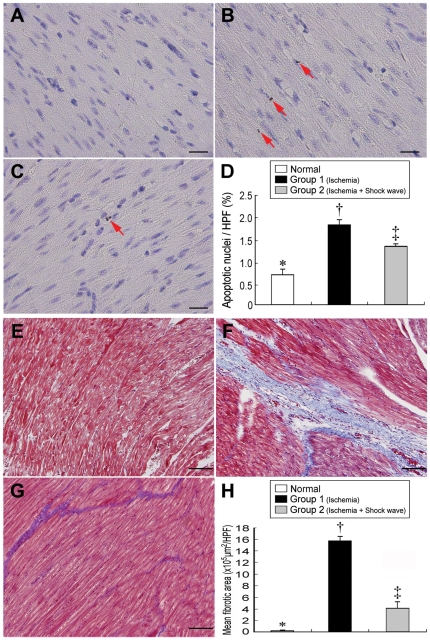
Immunohistochemical staining of fibrosis and apoptotic nuclei in LV myocardium. **Upper Panel:** TUNEL assay (400x) showing notably higher number of apoptotic nuclei (red arrows) after ischemic insult (**B**) compared with normal controls (**A**) and ischemia following shock wave treatment (**C**). †p<0.001 between the indicated groups (**D**). Scale bars in right lower corner represent 20 µm. n = 6 in each group. **Lower Panel:** Masson's Trichrome staining (100×) showing remarkably higher fibrotic area (blue) following ischemia (**F**) than in normal controls (**E**) and ischemia with shock wave treatment (**G**). †p<0.0001 between the indicated groups (**H**). Scale bars in right lower corner represent 100 µm. n = 6 in each group.

TUNEL assay (Fig. 3, A–D) identified notably higher number of apoptotic nuclei in group 1 than that in both normal controls and group 2, and remarkably higher in group 2 than in normal controls (all p<0.001). Furthermore, Masson's trichrome staining (Fig. 3, E–H) showed markedly increased fibrotic area in group 1 compared with that in normal controls and group 2, and significantly higher in group 2 than in normal controls (all p<0.0001). These findings may suggest that ECSW therapy attenuated cardiomyocyte death and myocardial fibrosis.

### Intensification of IHF and IHC Staining

**Figure 4:**
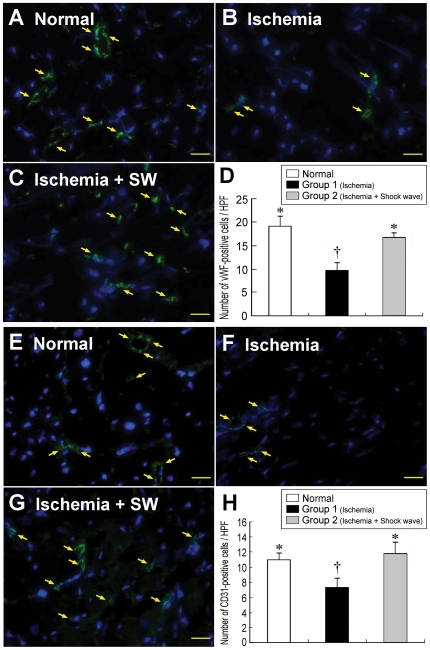
Immunofluorescent staining of vWF and CD31 in LV myocardium. **Upper Panel:** Immunofluorescence staining (400x) showing significantly lower number of vWF-positive cells (yellow arrows) after ischemia (**B**) than in normal controls (**A**) and ischemia with shock wave treatment (**C**). †p<0.001 between the indicated groups (**D**)**.** Scale bars in right lower corner represent 20 µm. **Lower Panel:** Immunofluorescence staining (400x) showing notably lower number of CD31-positive cells (yellow arrows) after ischemia (**F**) than in normal controls (**E**) and ischemia with shock wave treatment (**G**). †p<0.01 between the indicated groups (**H**). Scale bars in right lower corner represent 20 µm.

**Figure 5:**
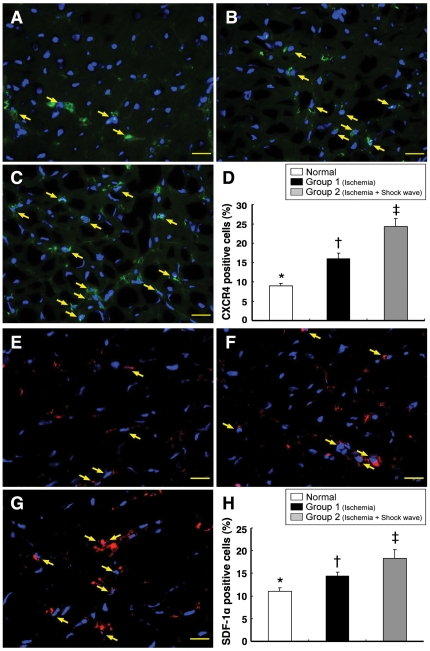
Immunofluorescent staining of CXCR4 and SDF-1α in LV myocardium. **Upper Panel:** Immunofluorescence staining (400x) showing significantly lower number of CXCR4-positive cells (yellow arrows) in normal controls (**A**) than in ischemic animals without (**B**) and with (**C**) shock wave treatment. *p<0.01 between the indicated groups (**D**). Scale bars in right lower corner represent 20 µm. **Lower Panel:** Immunofluorescence staining (400x) showing significantly lower number of SDF-1α-positive cells (yellow arrows) in normal controls (**E**) compared with animals after ischemia without (**F**) and with (**G**) shock wave treatment. *p<0.01 between the indicated groups (**H**). Scale bars in right lower corner represent 20 µm.

**Figure 6:**
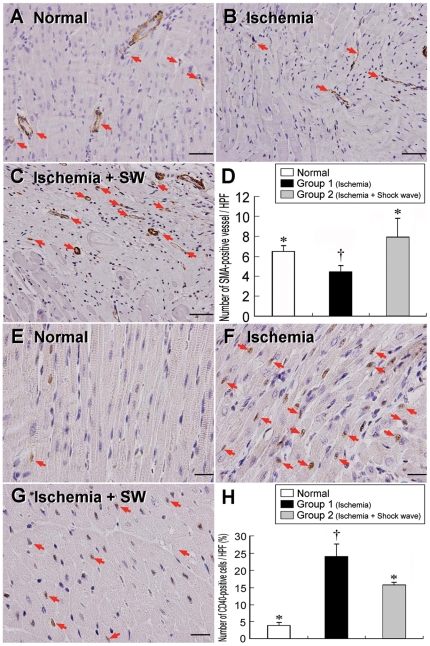
Immunohistochemical staining of α-SMA and CD40 nuclei in LV myocardium. **Upper panel:** α-SMA immunohistochemical staining (200x) showing notably lower number of small vessel (red arrows) after ischemia (**B**) than in normal controls (**A**) and ischemia with shock wave treatment (**C**). †p<0.002 between the indicated groups (**D**). Scale bars in right lower corner represent 50 µm. **Lower Panel:** IHC staining (400x) showing the CD40-positively stained cells were remarkably higher after ischemia (**F**) than in normal controls (**E**) and ischemia with shock wave treatment (**G**). †p<0.001 between the indicated group (**H)**. Scale bars in right lower corner represent 20 µm.

The results of IHF staining demonstrated that the numbers of CXCR4-, SDF-1α-, vWF-, and CD31-positive cells (Fig. 4 and 5) were remarkably higher in group 2 than in both normal controls and group 1 in LV myocardium (all p<0.001). Besides, the numbers of vWF- and CD31-positive cells (Fig. 4) were significantly higher in normal controls than in group 1 (all p<0.01). In contrast, the numbers of both CXCR4- and SDF-1α-positive cells (Fig. 5) were notably higher in group 1 than in normal controls (all p<0.01). IHC staining revealed that the number of small vessel (≤25 µm in diameter) (Fig. 6, A–D) was notably higher in group 2 than in both normal controls and group 1, and was significantly higher in normal controls than in group 1 (all p<0.002). On the other hand, IHC staining demonstrated that the number of CD40-positive cells (Fig. 6, E–H), an index of inflammation, was notably higher in group 1 than that in normal controls and group 2, and was significantly higher in group 2 than normal controls (all p<0.001). These findings indicate that ECSW therapy, in addition to enhancing angiogenesis, was effective in attenuating inflammatory response.

### mRNA Expression Patterns of Chemokines, Inflammatory Mediators, and Apoptotic Indexes in LV Myocardium

**Figure 7:**
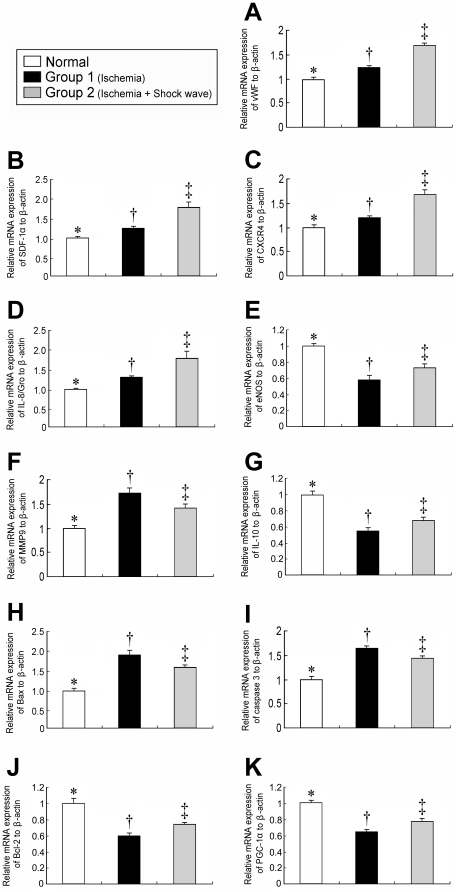
Profiles of mRNA expression in LV myocardium. **1**) Notably higher mRNA expressions of vWF (**A**), SDF-1α (**B**), CXCR4 (**C**) and interleukin (IL)-8/Gro (**D**) in ischemic animals with shock wave treatment compared with other groups. ‡p<0.01 between indicated groups. **2)** Remarkably lower mRNA expressions of eNOS (**E**) and IL-10 (**G**) after ischemia compared with other groups. †p<0.03 between indicated groups. 3) Significantly higher mRNA expressions of matrix metalloproteinase (MMP)-9 (**F**), Bax (**H**)**,** and caspase 3 (**I**) after ischemia than in other groups. †p<0.04 between indicated groups. Notably higher mRNA expressions of Bcl-2 (**J**) and PGC-1α (**K**) in animals after ischemic insult with shock wave treatment compared to those without. †p<0.02 between indicated groups.

The mRNA expressions of vWF, SDF-1α, and CXCR4 (Fig.7, A–C) were notably higher in group 2 than in controls and group 1, and significantly higher in group 1 than in normal controls (all p<0.01). In addition, mRNA expression of IL-8/Gro (Fig. 7D), an EPC-homing chemokine, was notably elevated in group 2 than in normal controls and group 1, and notably higher in group 1 than in normal controls (all p<0.01).

NO is the molecule related to angiogenesis and anti-inflammation. The mRNA expression of eNOS (Fig. 7E), an index of NO production, was remarkably suppressed in group 1 compared with that in normal controls and group 2, and was notably lower in group 2 than that in normal controls (all p<0.03). Moreover, the mRNA expression of MMP-9 (Fig. 7F), a pro-inflammatory index, was significantly elevated in group 1 than that in normal controls and group 2, and notably higher in group 2 than in normal controls (all p<0.04). On the other hand, the mRNA expression of IL-10 (Fig. 7G), an anti-inflammatory index, was notably lower in group 1 than in normal controls and group 2, and significantly lower in group 2 than normal controls (all p<0.03). These findings suggest that ECSW therapy exerted an anti-inflammatory effect.

The mRNA expressions of Bax (Fig. 7H) and caspase 3 (Fig. 7I), two indicators of apoptosis, were substantially higher in group 1 than in normal controls and group 2, and significantly higher in group 2 than in normal controls (all p<0.02). Conversely, the mRNA expression of Bcl-2 (Fig. 7J), an index of anti-apoptosis, was notably lower in group 1 than in normal controls and group 2, and significantly lower in group 2 than in normal controls (all p<0.02). These findings imply that ECSW therapy was anti-apoptotic.

The mRNA expression of PGC-1α (Fig. 7K), a transcriptional coactivator for upstream regulator of lipid catabolism, oxidative metabolism, mitochondrial metabolism and biogenesis, was notably lower in group 1 than in both normal controls and group 2, and significantly lower in group 2 than in normal controls (all p<0.02). This finding implicates ECSW therapy reversed the downregulation in PGC-1α gene expression in ischemia heart disease.

## Discussion

This study, which investigated the impact of ECSW on ischemia-related LV dysfunction, provided several striking implications. First, ECSW therapy markedly increased EPCs and EPC homing-related chemokines in LV ischemic area, thereby enhancing angiogenesis. Second, ECSW therapy remarkably reduced inflammatory response, oxidative stress, cellular apoptosis, and fibrotic change in ischemic LV myocardium. Third, of importance is the fact that ECSW substantially reversed ischemia-related LV dysfunction and significantly attenuated ischemia-induced LV remodeling without any side effect.

### ECSW Therapy Improves Heart Function and Inhibits LV Remodeling

Interestingly, while both clinical and experimental studies have clearly shown that ECSW therapy improved recovery of chronic hind limb ischemia and accelerated the healing process of ischemic skin ulceration [24], [25], [26], the effect of ECSW therapy on ischemia-related LV dysfunction has seldom been reported [15], [27]. The most important finding in the current study is that ECSW treatment significantly reversed LV dysfunction and LV remodeling. Besides, it is a non-invasive procedure with absolute safety as demonstrated in the presented study. Practically, the treatment for patients with severe diffuse CAD [12], [13], so-called “end-stage coronary artery disease”, remains a vexing problem for interventional cardiologists and surgeons over several decades because the majority of these patients are not suitable candidates for either PCI or CABG. The results of our study, in addition to supporting those from recent clinical observational studies [28], [29], also highlight the therapeutic potential of ECSW for patients with severe diffuse-obstructive CAD unsuitable for PCI and CABG.

### Mechanism of ECSW Therapy for Improving Heart Function—Enhancement of Angiogenesis

One previous animal study has revealed that application of ECSW in ischemic heart disease induced by restricting blood flow over left circumflex artery improved ischemia-related LV dysfunction through enhancing angiogenesis [15]. Besides, another recent study using a myocardial ischemia-reperfusion injury model in pigs has demonstrated that ECSW therapy enhanced protein expressions of eNOS and VEGF in ischemic myocardium [27]. However, evidence of angiogenesis after ECSW therapy was only supported by observation of an increased eNOS and VEGF in myocardium and angiographic findings of increased capillary density in that study [15], [27]. Therefore, the precise mechanism underlying ECSW-induced angiogenesis is still unclear [15]. One important finding in the present study is that the mRNA expressions of vWF, CXCR4, SDF-1α, eNOS, and IL-8/Gro were remarkably higher in group 2 (Constrictor over LAD plus ECSW) as compared with group 1 (Constrictor over LAD only). In addition, the protein expressions of VEGF, SDF-1α and CXCR4 were also notably higher in group 2 than in group 1. Moreover, IHF staining showed that the number of SDF-1α-, vWF-, CXCR4-, and CD31-positive cells were substantially higher in group 2 than in group 1. Furthermore, IHC staining for α-SMA demonstrated that the number of small vessel in LV myocardium was significantly higher in group 2 than in group 1. Our findings, in addition to reinforcing and extending the findings of the previous studies [15], [27], also support that angiogenesis may contribute to improved LV function after ECSW treatment.

### Mechanism of ECSW Therapy for Improving Heart Function—Attenuating Inflammatory Response, Oxidative Stress, and Cellular Apoptosis

The association among inflammation, reactive oxygen species, and cellular apoptosis/death has been fully investigated in the setting of myocardial ischemia [30], [31], [32]. An important finding in the present study is the notably lower mRNA expression of MMP-9 and the remarkably elevated mRNA expression of IL-10 in LV myocardium after ECSW treatment on day 180 after the procedure. Furthermore, the significantly reduced oxidative stress and the suppression of CD40-positive cells in LV myocardium after ECSW administration imply significant roles of ECSW in attenuating inflammation and oxidative stress in ischemic myocardium.

Importantly, ECSW therapy also remarkably reduced the mRNA expressions of Bax and caspase 3 and significantly increased the mRNA expression of Bcl-2. The anti-apoptotic action of ECSW was further confirmed by TUNEL staining of LV myocardium. Furthermore, ECSW treatment also significantly reduced fibrosis of LV myocardium. Of importance is that ECSW therapy significantly preserved the level of PGC-1α gene expression and mitochondrial cytochrome C and maintained the protein expression of Cx43 in LV myocardium which have been shown to be depressed in the setting of dilated cardiomyopathy [33]. These findings further strengthen the therapeutic potential of ECSW in reversing ischemia-related LV dysfunction and remodeling through inhibiting cellular apoptosis/death and myocardial fibrosis as well as preserving the integrities of Cx43 and energy biogenesis and metabolism which plays a key role in electrical coupling between cardiomyocytes [34], [35].

**Figure 8:**
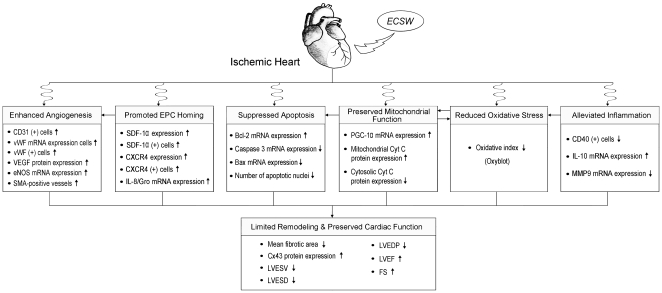
The proposed mechanisms. Proposed mechanisms of extra-corporeal shock wave therapy on improving left ventricular (LV) function and attenuating LV remodeling in a porcine model of ischemic heart disease.

In conclusion, ECSW therapy significantly reversed ischemia-related LV remodeling and preserved LV function through enhancing angiogenesis, attenuating inflammation, and suppressing oxidative stress. The proposed mechanisms of potential impacts of ECSW therapy on preserving heart function in the porcine model have been summarized in Figure 8.

### Study Limitations

This study has limitations. First, since serial studies on the cause-and-effect relationship using different dosages of ECSW have not been performed, the optimal dosage for maximal therapeutic effects within safety margin is still unknown. Additionally, the sample size of the current study was relatively small. However, the impact of our results, on the other hand, is impressive. Second, this study did not investigate in details the mechanistic basis of ECSW therapy underlying the observed pro-angiogenic, anti-inflammatory, and anti-oxidant effects at molecular-cellular level. Further studies, therefore, may be warranted to clarify a cause-and-effect relationship when it comes to mechanistic findings of ESCW treatment against ischemic cardiac dysfunction and remodeling. Third, this study did not examine the effect of ECSW therapy on normal mini-pig myocardium. Thus, we did not provide information on the possibility of enhancement of angiogenesis through ECSW on normal mini-pig myocardium.
